# Open vs. Laparoscopic Surgery for Rectal Cancer: Impact on Identification and Preservation of Pelvic Autonomic Nerves and Effects on Urinary and Sexual Function and Quality of Life

**DOI:** 10.3390/jcm15062421

**Published:** 2026-03-21

**Authors:** Ivan Dimitrijevic, Marko Miladinov, Jelenko Jelenkovic, Predrag Gavrilovic, Aleksandar Sekulic, Goran Barisic, Jovana Rosic Stojkovic

**Affiliations:** 1 Faculty of Medicine, University of Belgrade, 11000 Belgrade, Serbia; ivanclean@gmail.com (I.D.); sekulic038@yahoo.com (A.S.); barisic_goran@yahoo.com (G.B.); 2 Clinic for Digestive Surgery—First Surgical Clinic, University Clinical Center of Serbia, 11000 Belgrade, Serbia; marko.miladinov90@gmail.com (M.M.); jelenkojelenkovic1@gmail.com (J.J.); 3 Center for Medical Informatics, 11000 Belgrade, Serbia; gavrilo@ymail.com

**Keywords:** rectal cancer, laparoscopic surgery, pelvic autonomic nerves, nerve preservation, urinary function, sexual function, quality of life

## Abstract

**Background/Objectives**: With advances in surgical techniques for rectal cancer—particularly laparoscopic and robotic-assisted approaches—the choice of operative method may influence not only oncological but also functional outcomes. This study aimed to compare open and laparoscopic rectal cancer surgery regarding pelvic autonomic nerve identification, preservation and its impact on postoperative urinary, sexual, and quality-of-life outcomes. **Methods**: A total of 181 patients who underwent curative rectal cancer surgery at the Clinic for Digestive Surgery, University Clinical Center of Serbia, were included. Six types of procedures were performed using both open and laparoscopic approaches. Intraoperative identification and preservation of pelvic autonomic nerves were assessed and verified postoperatively through evaluation of urinary, sexual, and quality-of-life parameters. Urinary function and related life quality were assessed using the International Prostate Symptom Score (IPSS and IPSS-QoL), while sexual function was evaluated using gender-specific validated questionnaires (IIEF-15 and FSFI) preoperatively and at 2, 4, and 6 months postoperatively. **Results**: Nerve non-visualization and/or injuries were significantly more frequent in the open surgery group. The laparoscopic approach was associated with better preservation of urinary function, particularly among male patients, better sexual function in both sexes, and a transient advantage in quality of life. **Conclusions**: Laparoscopic rectal cancer surgery demonstrated superior pelvic autonomic nerve visualization and preservation and better short-term urinary and sexual function. Nonetheless, quality-of-life outcomes were comparable by 6 months of follow-up, underscoring the importance of meticulous nerve-preserving technique regardless of surgical approach.

## 1. Introduction

Surgical resection remains the cornerstone of curative therapy in rectal cancer. The introduction of the total mesorectal excision (TME) technique, characterized by meticulous anatomical dissection along the pelvic fascia with careful identification and preservation of vascular and autonomic nerve structures, has led to major improvements in oncological outcomes, particularly by reducing local recurrence rates and better preserving pelvic organ function [1,2,3]. In parallel, neoadjuvant chemoradiotherapy (nCRT) has become the standard treatment for locally advanced rectal cancer, often followed by adjuvant chemotherapy, while total neoadjuvant therapy (TNT) has emerged as an alternative preoperative strategy [4,5,6,7]. Among surgical innovations, minimally invasive techniques—particularly laparoscopy and, more recently, robotic-assisted surgery—have gained wide acceptance, with large, randomized trials confirming oncological outcomes comparable to open resection [8,9,10]. Nevertheless, open surgery remains indispensable in cases of locally advanced or bulky tumors requiring multivisceral resection, anatomically complex situations such as obesity, narrow pelvis, or adhesions, and when minimally invasive approaches are contraindicated or not technically feasible [11,12].

Encouraged by these advances, the focus of research has progressively expanded beyond tumor eradication to include the preservation of patients’ quality of life [13]. Pelvic organ function is now regarded as an important endpoint alongside traditional outcomes such as survival, recurrence, and perioperative morbidity, encompassing urinary, sexual, and health-related quality-of-life domains, while the choice of surgical approach can influence patient outcomes beyond oncological considerations. This study aimed to evaluate differences between open and laparoscopic rectal cancer surgery in the identification and preservation of pelvic autonomic nerves, as well as to compare their effects on postoperative urinary and sexual function and quality of life.

## 2. Materials and Methods

### 2.1. Patient Cohort

This retrospective comparative study included 181 rectal cancer patients treated at the Clinic for Digestive Surgery—First Surgical Clinic, University Clinical Center of Serbia, between April 2008 and February 2020. Patients aged ≥ 18 years with primary sporadic rectal adenocarcinoma located ≤15 cm from the anal verge who underwent open or laparoscopic surgery were eligible for inclusion. Patients were excluded if they were younger than 18 years, had benign or recurrent rectal lesions, familial adenomatous polyposis or hereditary nonpolyposis colorectal cancer, underwent non-elective surgery, had incomplete clinical data, a history of previous pelvic surgery, or a preexisting urinary or sexual dysfunction. Cases converted from laparoscopy to open surgery were excluded, as conversion was usually due to unexpected intraoperative findings or adverse events that could confound functional outcomes. Ethical approval for this study was obtained from the Ethics Committee of the University Clinical Center of Serbia (approval number 1600/30, 13 October 2025), and the study was conducted in accordance with the principles of the Declaration of Helsinki. A waiver of informed consent was granted, as this was a retrospective observational study using preexisting, de-identified clinical records that posed minimal risk to participants and made obtaining individual consent unfeasible.

### 2.2. Surgical Interventions

All included patients underwent rectal cancer surgery, with six different procedures performed using either open or laparoscopic approaches: low anterior resection (LAR) with partial mesorectal excision, LAR with total mesorectal excision, LAR with intersphincteric dissection, Miles, Miles–Thompson, and Hartmann’s procedures. The choice between open and laparoscopic surgery was made preoperatively based on tumor characteristics, patient-related factors, and surgeon experience. Laparoscopic surgery was preferentially performed in patients with favorable anatomy, non-bulky tumors, and without suspected multivisceral involvement. All procedures were performed by the same specialized surgical team at a high-volume center; laparoscopic rectal surgery was gradually introduced during the early study period and initially reserved for carefully selected patients, with indications expanding as institutional experience increased.

### 2.3. Outcome Measures

During surgery, pelvic autonomic nerves were assessed intraoperatively based on the surgeon’s visual inspection and operative judgment and categorized as either visualized or not visualized and/or injured. The latter category included cases in which nerves could not be clearly identified or were suspected to be injured during dissection. This assessment was qualitative and based on operative reports. These findings were further evaluated postoperatively through standardized questionnaires addressing urinary function and life quality, as well as male and female sexual function. Patients completed the questionnaires preoperatively and at 2, 4, and 6 months after surgery, allowing monitoring of functional outcomes over time.

#### 2.3.1. Urinary Function and Quality of Life

Urinary function in this study was assessed using the International Prostate Symptom Score (IPSS) [14], a validated questionnaire widely applied in both men and women [15,16,17], and previously used in patients undergoing rectal cancer surgery [17,18,19]. The IPSS consists of seven items addressing incomplete emptying, frequency, intermittency, urgency, weak stream, straining, and nocturia. Each item is scored from 0 to 5, yielding a total score between 0 and 35. According to the American Urological Association classification, total scores are categorized as mild (0–7), moderate (8–19), or severe (20–35) urinary symptoms. In addition, the IPSS quality of life (IPSS-QoL) score was used to evaluate the impact of urinary symptoms on daily living. This complementary single-item measure ranges from 0 (“delighted”) to 6 (“terrible”), with higher values reflecting a greater adverse effect on quality of life.

#### 2.3.2. Male Sexual Function

The International Index of Erectile Function (IIEF-15) questionnaire was used to evaluate male sexual function. The IIEF-15 is a validated multidomain instrument that assesses five domains of sexual function: erectile function (6 items; range 6–30), orgasmic function (2 items; range 0–10), sexual desire (2 items; range 2–10), intercourse satisfaction (3 items; range 0–15), and overall satisfaction (2 items; range 2–10) [20]. Higher scores in each domain indicate better sexual function. The erectile function domain is most frequently used in clinical practice as the primary outcome measure, with erectile dysfunction severity classified according to established criteria as severe (6–10), moderate (11–16), mild-to-moderate (17–21), mild (22–25), and no erectile dysfunction (26–30) [21].

#### 2.3.3. Female Sexual Function

The Female Sexual Function Index (FSFI) was used to evaluate female sexual function in this study [22]. The FSFI is a validated 19-item self-report questionnaire that assesses six domains of sexual function: desire, arousal, lubrication, orgasm, satisfaction, and pain. Each item is scored on a Likert scale, and domain scores are obtained by summing relevant items and multiplying by a domain-specific factor. The resulting domain scores are then combined to generate a total FSFI score ranging from 2 to 36, with higher scores indicating better sexual function. A total FSFI score of 26.55 or lower is commonly used as a cutoff for diagnosing female sexual dysfunction.

### 2.4. Statistical Analysis

Univariate, propensity score, and multivariate analyses were performed using Statistica software, version 13.5.0.17 (TIBCO Software Inc., Palo Alto, CA, USA), and R (version 4.2.2; RStudio interface), while graphical visualization was conducted using GraphPad Prism, version 8.4.3 (GraphPad Software Inc., San Diego, CA, USA). In the univariate analyses, due to the shape of the data distribution and the size of the analyzed samples, nonparametric statistical methods were applied. The Mann–Whitney U test (MW) was applied for comparisons between two independent groups. For repeated measures of continuous or ordinal variables, the Friedman nonparametric analysis of variance by ranks was employed (ANOVA). Categorical variables were analyzed using Pearson’s chi-square test (χ^2^) when expected frequencies were adequate; otherwise, the two-tailed Fisher’s exact test (FT) was employed. Results are presented with corresponding *p*-values, and values < 0.05 were considered statistically significant.

To address baseline imbalances between surgical groups, propensity score (PS) analysis was conducted. The PS model included clinically relevant covariates (age, neoadjuvant and adjuvant therapy variables, tumor height and volume, pathological T and N stage, and preoperative functional scores). Propensity scores were estimated using generalized boosted models (GBM), and full matching was applied. Covariate balance was assessed using standardized mean differences (SMD), with absolute values < 0.10 considered indicative of adequate balance.

To evaluate the simultaneous effect of surgical technique across functional domains, multivariate analysis of covariance (MANCOVA) was performed separately at each postoperative time point, with surgical approach as the factor and baseline values as covariates. Four multivariate statistics were calculated (Pillai’s trace, Wilks’ lambda, Hotelling–Lawley trace, and Roy’s largest root), with primary interpretation based on Pillai’s trace. Bonferroni correction was applied for comparisons across three postoperative time points.

All tests were two-sided, and *p*-values < 0.05 were considered statistically significant.

## 3. Results

Baseline clinicopathological characteristics of patients and the comparison between open and laparoscopic surgery groups are presented in Table 1.

**Figure 1:**
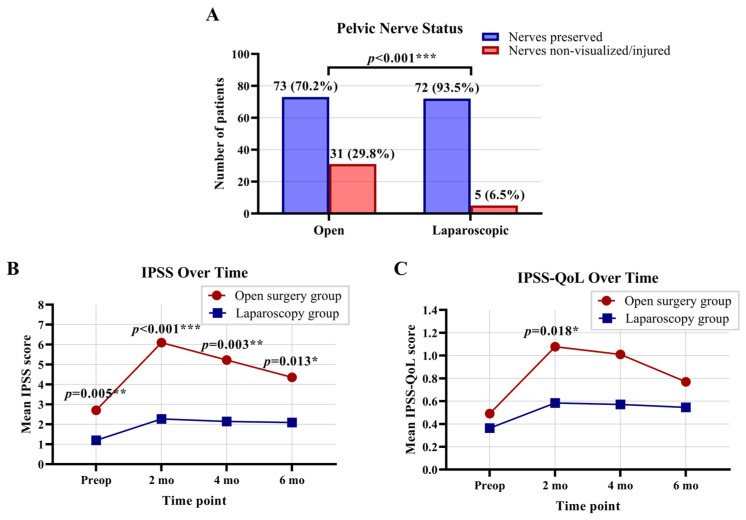
Comparison of pelvic autonomic nerve preservation between open and laparoscopic surgery and its impact on postoperative urinary function and quality of life assessed preoperatively (preop) and at 2, 4, and 6 months (mo) postoperatively. (**A**) Nerve identification and preservation. (**B**) Urinary function (International Prostate Symptom Score, IPSS). (**C**) Quality of life (IPSS-QoL). Groups and exact *p*-values are shown in the graphs. Significant differences are also indicated by asterisks (*p* < 0.05 *, *p* < 0.01 **, *p* < 0.001 ***); two-tailed Fisher’s exact test, Mann–Whitney U test.

Non-visualization and/or injury of the pelvic autonomic nerves occurred in 36 (20%) patients overall. A significant difference between the open and laparoscopic approaches in the identification and preservation of pelvic autonomic nerves is shown in Figure 1A. Significant association was observed between the type of surgical technique and the occurrence of non-visualization/injury (χ^2^(5) = 21.10, *p* < 0.001; χ^2^). Non-visualization/injury was also more common in male patients and in those who received preoperative therapy (*p* = 0.022 and *p* = 0.025, respectively; two-tailed FT for both). Postoperative chemotherapy was administered in 30% of patients with non-visualization/injury, compared with 12% of those without, a difference that was statistically significant (*p* = 0.003; two-tailed FT).

Differences in IPSS and IPSS-QoL scores between the open surgery and laparoscopy groups at each time point are presented in Figure 1B,C. Regarding urinary function and life quality in male patients, a significant difference in IPSSs was observed between the laparoscopy and open surgery groups preoperatively, as well as at 2, 4, and 6 months postoperatively (*p* = 0.012, *p* < 0.001, *p* = 0.001, and *p* = 0.004, respectively; MW). Conversely, IPSS-QoL scores showed no difference preoperatively, but differed significantly at 2 and 4 months postoperatively (*p* < 0.017 and *p* = 0.020; MW), before showing no significant difference again at 6 months. When evaluating urinary function and quality of life in female patients, no significant differences in IPSS or IPSS-QoL scores were observed between the laparoscopic and open surgery groups at any time point.

**Figure 2:**
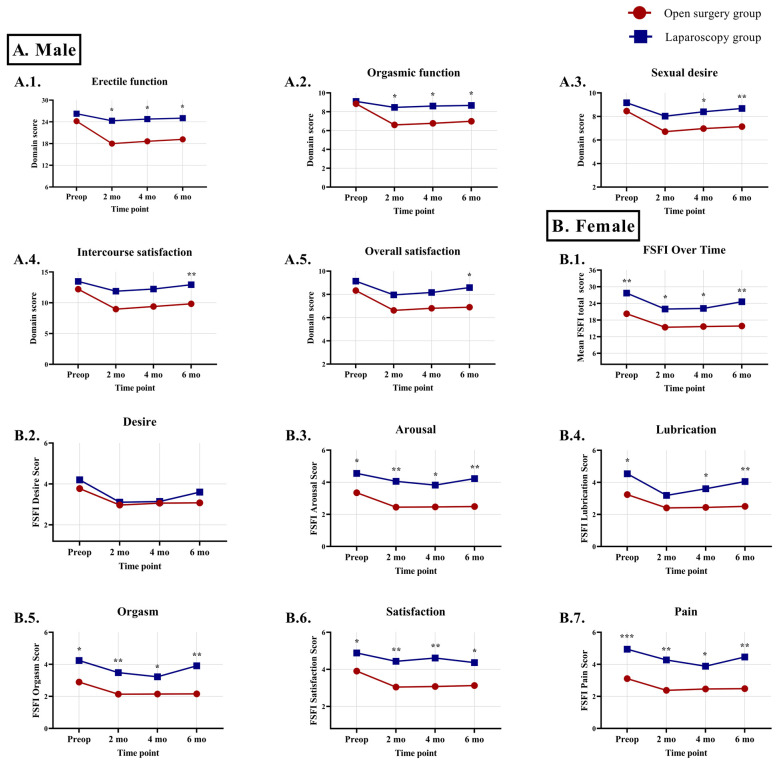
Comparison of male and female sexual function between open and laparoscopic surgery, assessed preoperatively (preop) and at 2, 4, and 6 months (mo) postoperatively. (**A.1.**–**A.5.**) All five domains of the International Index of Erectile Function (IIEF-15) are presented for men. (**B.1.**–**B.7.**) The Female Sexual Function Index (FSFI) total score and all six FSFI domains are presented for women. Significant differences between open and laparoscopic surgical approaches are indicated by asterisks (* *p* < 0.05, ** *p* < 0.01, *** *p* < 0.001; Mann–Whitney U test).

In men, greater postoperative erectile dysfunction was observed in the open surgery group, with differences across all five IIEF-15 domains illustrated in Figure 2(A.1.–A.5.). In women, the open surgery group demonstrated greater sexual dysfunction already preoperatively, and this pattern persisted across postoperative time points, as reflected by the FSFI total score and five of the six FSFI domains (Figure 2(B.1.–B.7.)).

Baseline characteristics demonstrated initial imbalances between open and laparoscopic surgery groups within each sex. Among men, significant differences were observed in age (*p* < 0.001), neoadjuvant therapy (*p* = 0.022), tumor height and volume (both *p* < 0.001), and pathological T and N stages (*p* = 0.002 and *p* = 0.022). Among women, significant differences were observed in neoadjuvant therapy (*p* = 0.038), tumor volume (*p* < 0.001), and pathological T stage (*p* = 0.008), supporting the use of covariate-adjusted propensity score analyses to control for potential confounding.

Covariate balance before and after GBM-based propensity score full matching was assessed using standardized mean differences and visualized using Love plots for the propensity score models evaluating urinary and sexual outcomes in men and women (Supplement Figure S1). Matching substantially improved the balance between surgical groups, with most covariates shifting toward zero standardized difference after adjustment, although some residual differences remained.

The adjusted effects of surgical approach on postoperative urinary and sexual outcomes, estimated using GBM-based propensity score full matching with Bonferroni correction, are presented in Table 2. Among men, significant differences between surgical approaches were observed in both urinary and sexual function domains at 2 and 4 months, with all sexual function domains remaining significant at 6 months. Among women, no differences were observed in urinary outcomes, while selected sexual function domains showed significant differences, although these effects were not uniform across time points.

To assess the global effect of surgical approach across all functional domains simultaneously, MANCOVA was performed at each postoperative time point. A significant multivariate effect was observed in both men and women at 2, 4, and 6 months (all *p* < 0.001; Supplement Table S1).

## 4. Discussion

In this retrospective analysis, laparoscopic rectal cancer surgery was associated with better short-term urinary and sexual function compared with open surgery, while quality-of-life outcomes were largely comparable during the 6-month follow-up. These findings suggest that minimally invasive surgery may offer functional advantages in the early postoperative period, potentially reflecting reduced pelvic trauma and improved preservation of autonomic nerve function.

Pelvic autonomic nerve damage often stems from poor identification, which is more challenging in the narrow male pelvis, and is influenced by tumor location and surgical approach [23]. Our study confirmed these findings, showing that nerve non-visualization/injury was associated with the type of surgery, occurred more frequently in male patients, and was more common among those who received preoperative therapy. Additionally, the laparoscopic approach was linked to better nerve visualization/preservation; however, this may partly reflect the higher proportion of low rectal tumors in the open surgery group, as lower tumor location is a well-recognized risk factor for pelvic autonomic nerve injury [24]. In low rectal cancers, the confined space increases the risk of combined injury to pelvic splanchnic and levator ani nerves, and additional damage can also result from inflammation, diathermy, sutures, or radiotherapy, which may induce demyelination, vascular injury, and fibrosis. The magnified visualization provided by laparoscopic surgery may facilitate identification of pelvic nerves and thereby contribute to improved postoperative functional outcomes [23]. However, achieving these benefits requires substantial surgical experience and technical proficiency. Current findings are heterogeneous, and no consistent advantage has been demonstrated for either laparoscopic or open surgery in preserving sexual or urinary function. [23,25]. Intraoperative identification/preservation of pelvic autonomic nerves is infrequently reported as a formal endpoint in comparative TME studies, although several neuromonitoring and anatomy-focused series have assessed it directly. Our inclusion of this parameter therefore represents a methodological strength of the present analysis.

Urinary dysfunction after rectal cancer surgery mainly results from nerves disruption. Sympathetic injury can provoke detrusor overactivity and urge incontinence, while impaired structural support—dependent on ligaments, connective tissue, levator ani, and estrogen-sensitive tissues—predisposes to stress incontinence. Parasympathetic damage can cause postoperative detrusor hypoactivity, reduced bladder sensation, and retention with overflow incontinence, which may be transient if partial nerve preservation allows regeneration. Persistent dysfunction beyond one year usually indicates irreversible bladder underactivity. Radiotherapy may induce fibrosis of the bladder and sphincters, though its contribution remains debated, with large trials suggesting surgical factors are the primary cause. In our study, a significant preoperative difference in urinary function between the open and laparoscopic groups was observed and persisted at 2, 4, and 6 months postoperatively in both univariate and multivariate analyses. However, propensity score analysis showed that these differences were primarily observed in male patients, particularly during the early postoperative period. A significant difference in urinary function and related quality of life between open and laparoscopic surgery was observed only in male patients, whereas no such difference was found among females. This finding suggests that preservation of the pelvic autonomic nerves is inherently more challenging in male patients due to their narrower and deeper pelvic anatomy. Consequently, pelvic dissection in men requires more refined total TME and meticulous nerve-sparing techniques, for which the robotic approach provides the greatest technical advantage [26].

Sexual dysfunction is multifactorial, encompassing psychological distress as well as direct neurovascular injury [27]. In men, sympathetic damage impairs ejaculation, and parasympathetic injury disrupts vasodilation of erectile tissue, leading to erectile dysfunction; radiotherapy adds vascular injury and can lower testosterone, further impairing function. Although the total IIEF-15 score has been used in some studies as a global indicator of sexual function, the present analysis was based on domain-specific scoring, in line with the original validation of the instrument [20,28,29]. In line with expectations, greater erectile dysfunction was observed at all postoperative time points in the open surgery group. Nevertheless, the progressive improvement in erectile function seen in both groups suggests a gradual recovery process, likely reflecting the resolution of postoperative pelvic tissue inflammation and partial regeneration of minor nerve injury caused by intraoperative traction or tension during dissection.

Although mechanisms of sexual dysfunction in women are less defined, sympathetic injury may reduce lubrication and orgasmic sensation, while parasympathetic damage may blunt genital swelling responses. Radiation can exacerbate sexual dysfunction by inducing vaginal atrophy, fibrosis, and ovarian failure, causing premature menopause, vaginal dryness, and dyspareunia. In our study, female sexual dysfunction was consistently greater at all evaluated time points in the open surgery group; however, propensity score analysis adjusting for potential confounders showed that these differences were less pronounced and not consistently sustained. The differential impact of surgical approach on urinary and sexual function in female patients may be attributed to the relative resilience of lower urinary tract mechanisms to mild autonomic injury and postoperative inflammation, whereas sexual function—being more dependent on delicate autonomic and vascular pathways—remains more susceptible to subtle pelvic nerve damage; thus, the observed superiority of laparoscopic over open surgery in preserving sexual function may partly reflect reduced tissue trauma, smaller scars, and faster recovery [30].

After rigorous adjustment using GBM-based propensity score full matching, laparoscopic surgery was associated with superior postoperative functional outcomes, particularly among men. The most pronounced differences were observed at early postoperative time points, suggesting a faster recovery of urinary and sexual function following the minimally invasive approach. Although several significant domain-specific differences were also identified in women, these effects were more variable over time. Importantly, the consistent multivariate significance across all time points supports a global effect of surgical technique on postoperative functional recovery.

The non-random allocation of patients to surgical approach, influenced by tumor characteristics, patient-related factors, and surgeon preference, represents an inherent limitation of this retrospective study and may introduce residual confounding despite statistical adjustment. Unmeasured factors such as body mass index, pelvic anatomical complexity, and prior abdominal surgery may have influenced both surgical selection and postoperative functional outcomes. Because follow-up was limited to 6 months, our findings primarily reflect short-term recovery, and longer-term functional outcomes—particularly sexual function—may not be fully captured.

## 5. Conclusions

Laparoscopic surgery was associated with improved postoperative urinary and sexual functional recovery compared with open surgery, particularly among men and during early follow-up. Although beneficial effects were also observed in several sexual function domains among women, these differences were less consistent over time. Taken together, these findings suggest that minimally invasive surgery may facilitate faster functional recovery, while overall quality-of-life outcomes appear comparable across the follow-up period.

## Figures and Tables

**Table 1:** Baseline characteristics and comparison of open and laparoscopic surgery groups.

Characteristic	Total (*n* = 181)	Open Surgery (*n* = 104)	Laparoscopy (*n* = 77)	Test/Statistic	*p*-Value
Sex				χ^2^(1) = 1.072	0.301
Male, *n* (%)	109 (60.2)	66 (63.5)	43 (55.8)		
Female, *n* (%)	72 (39.8)	38 (36.5)	34 (44.2)		
Age (years), mean ± SD	56 ± 11	53 ± 8	59 ± 12	MW	<0.001
Tumor height (cm), mean ± SD	8.59 ± 4.03	7.63 ± 3.64	9.90 ± 4.19	MW	<0.001
T stage				MW	0.126
0 *	9 (4.9)	0 (0)	9 (11.7)		
1	7 (3.9)	7 (3.9)	7 (9.1)		
2	42 (23.2)	33 (31.7)	9 (11.7)		
3	94 (51.9)	51 (49)	43 (55.8)		
4	24 (13.3)	16 (15.4)	8 (10.4)		
Tis	5 (2.8)	4 (3.8)	1 (1.3)		
N stage				MW	0.058
0	106 (58.5)	56 (53.8)	50 (64.9)		
1	45 (24.9)	23 (22.1)	22 (28.6)		
2	30 (16.6)	25 (24.0)	5 (6.5)		
M stage				MW	0.185
0	169 (93.4)	92 (88.5)	77 (100)		
1	12 (6.6)	12 (11.5)	0 (0)		
Operative technique, *n* (%)				χ^2^(5) = 18.855	0.002
LAR with partial mesorectal excision	76 (42.0)	34 (32.7)	42 (54.5)		
LAR with total mesorectal excision	53 (29.3)	33 (31.7)	20 (26.0)		
LAR with intersphincteric dissection	19 (10.5)	15 (14.4)	4 (5.2)		
Miles	13 (7.2)	11 (10.6)	2 (2.6)		
Miles–Thompson	12 (6.6)	4 (3.8)	8 (10.4)		
Hartmann	8 (4.4)	7 (6.7)	1 (1.3)		
Neoadjuvant chemoradiotherapy, *n* (%)	55 (30.4)	22 (21.2)	33 (42.8)	FT	0.002
Postoperative chemotherapy, *n* (%)	80 (44.2)	48 (46.2)	32 (41.6)	FT	0.549

* Patients who initially underwent endoscopic polypectomy with histologically confirmed adenocarcinoma and inadequate resection margins and subsequently underwent definitive surgical resection.

**Table 2:** Adjusted effect of surgical approach on urinary and sexual outcomes estimated using GBM-based propensity score full matching (Bonferroni-corrected *p*-values).

Sex	Function	Outcome	2 Months	4 Months	6 Months
Male	Urinary	IPSS	0.014	0.023	0.232
		IPSS-QoL	<0.001	0.005	0.068
	Sexual	Erectile function	0.028	0.026	0.003
		Orgasmic function	0.001	0.001	0.002
		Sexual desire	0.003	0.003	0.001
		Intercourse satisfaction	0.005	0.005	0.012
		Overall satisfaction	0.020	0.009	0.007
Female	Urinary	IPSS	1	1	1
		IPSS-QoL	0.621	1	1
	Sexual	FSFI	0.015	0.069	0.011
		Desire	0.117	0.167	0.026
		Arousal	0.716	0.208	0.033
		Lubrication	0.077	0.259	0.015
		Orgasm	0.004	0.092	0.003
		Satisfaction	1	1	1
		Pain	0.032	0.012	0.085

## Data Availability

The data presented in this study are available on request from the corresponding author. Due to the presence of sensitive patient information, the dataset cannot be made publicly accessible to protect participant confidentiality and to comply with institutional and ethical regulations.

## Supplementary Materials

The following supporting information can be downloaded at: https://www.mdpi.com/article/10.3390/jcm15062421/s1. Supplement Figure S1 Love plots showing standardized mean differences (SMDs) for covariates before and after GBM-based propensity score full matching in men (A) and women (B) for urinary (1) and sexual (2) function outcomes. The vertical dashed lines indicate the conventional threshold of 0.10 for acceptable covariate balance. Supplement Table S1 Multivariate analysis of covariance (MANCOVA) assessing the simultaneous effect of surgical approach on all functional domains at 2, 4, and 6 months postoperatively, stratified by sex.
