# Supplementary material for: Open vs. Laparoscopic Surgery for Rectal Cancer: Impact on Identification and Preservation of Pelvic Autonomic Nerves and Effects on Urinary and Sexual Function and Quality of Life
Source: J Clin Med. 2026 Mar 21;15(6):2421. doi: 10.3390/jcm15062421 (PMC13027352; doi:10.3390/jcm15062421)
Supplement: Supplementary file 1 [file jcm-15-02421-s001.zip › jcm-4121105-supplementary.pdf]

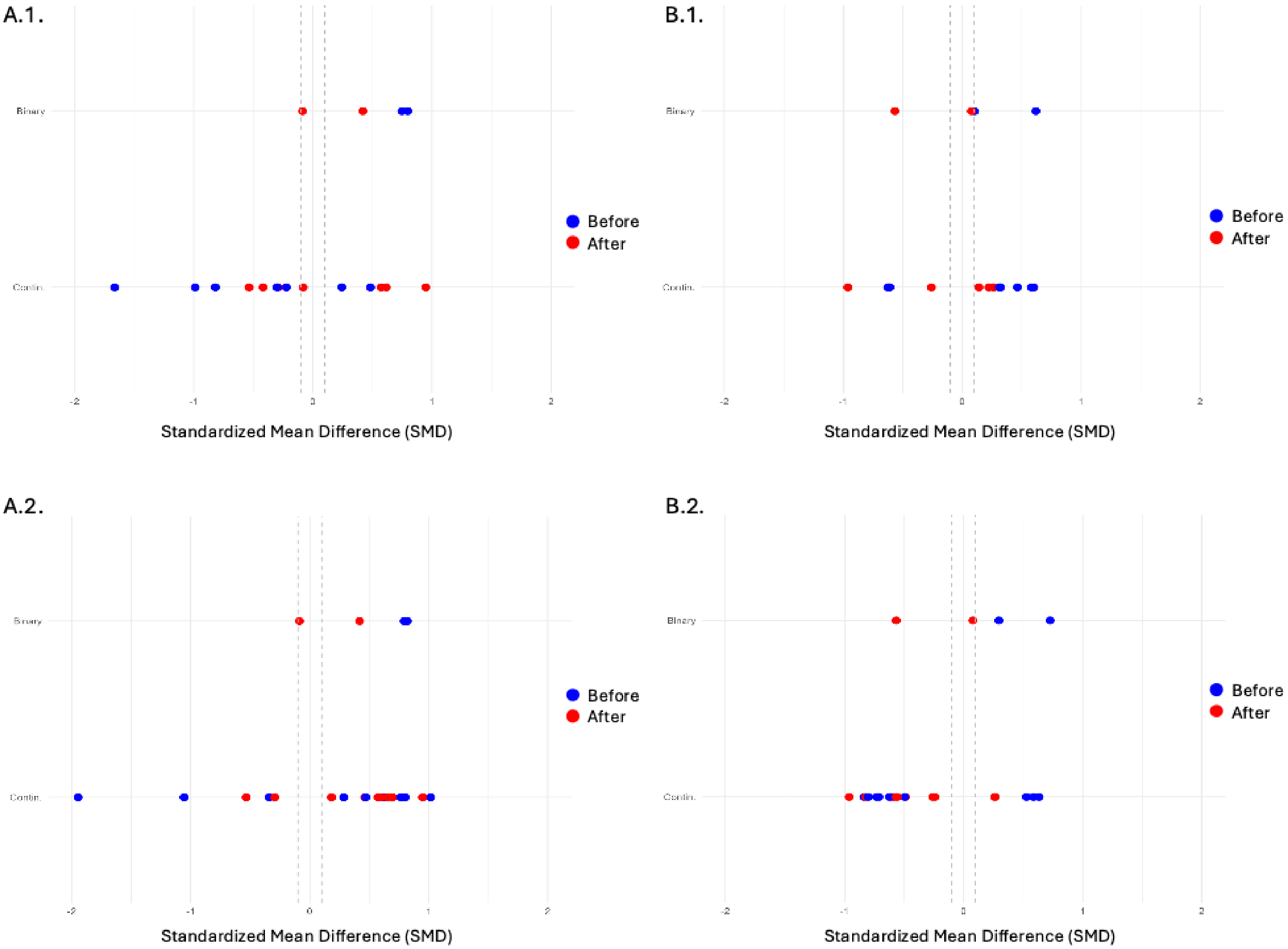

Supplement Figure S1. Love plots showing standardized mean differences (SMDs) for covariates before and after GBM-based propensity score full matching in men (A) and women (B) for urinary (1) and sexual (2) function outcomes. The vertical dashed lines indicate the conventional threshold of 0.10 for acceptable covariate balance.

Supplement Table S1. Multivariate analysis of covariance (MANCOVA) assessing the simultaneous effect of surgical approach on all functional domains at 2, 4, and 6 months postoperatively, stratified by sex.

Sex	Time	Pillai's trace	n	F	df1	df2	p-value
Men	2 months	0.394	109	8.078	7	87	<0.001
Men	4 months	0.289	109	5.058	7	87	<0.001
Men	6 months	0.302	109	5.365	7	87	<0.001
Women	2 months	0.796	72	19.907	9	46	<0.001
Women	4 months	0.703	72	12.105	9	46	<0.001
Women	6 months	0.639	72	9.041	9	46	<0.001

Models adjusted for baseline functional values. Pillai's trace is reported as the primary multivariate statistic.
